# Large single cutaneous metastasis of colon adenocarcinoma mimicking a squamous cell carcinoma of the skin: A case report

**DOI:** 10.1016/j.ijscr.2019.02.043

**Published:** 2019-03-07

**Authors:** Mario Faenza, Giuseppe Del Torto, Pasquale Di Costanzo, Gorizio Pieretti, Rossella Lamberti, Renato Franco, Giuseppe A. Ferraro, Giovanni F. Nicoletti

**Affiliations:** a Multidisciplinary Department of Medical and Dental Specialties, Università degli Studi della Campania "Luigi Vanvitelli", Plastic Surgery Unit, Naples, Italy; b Department of Mental and Physical Health and Preventive Medicine, Università degli Studi della Campania "Luigi Vanvitelli", Pathology Unit, Naples, Italy

**Keywords:** Skin cancer, Cutaneous Metastasis, Colon Cancer, Fasciocutaneous Flap

## Abstract

**Introduction:**

Metastases represent one of the most outstanding characteristics of malignant neoplasms and are relatively rare in the skin, in spite of the great extension of the cutaneous organs.

Development of cutaneous metastases from colon cancer is a rare event, usually occurring in widely disseminated disease and commonly leading to a poor prognosis.

As to location, cutaneous metastases often favor areas close to the primary malignancy, such as lung cancer and skin metastases on the trunk. However, remote sites as the scalp may be also involved.

**Case presentation:**

We present the case of a 92-year-old female patient with a massive single nodular skin lesion on her left supraclavicular area, that came back positive for cutaneous metastasis of colon adenocarcinoma.

**Discussion:**

Cutaneous metastasis of colorectal cancer a rare event (2.3%–6%) that usually occur two years after the detection or resection of the primary tumor. It seldom occurs before the identification of the primary tumor and involvement of secondary organs, such as the liver. There are few cases reported with only cutaneous metastases.

**Conclusion:**

In conclusion, dermatological evaluation of patients who are undergoing screening or who have already been diagnosed with cancer is extremely important.

## Highlights

• Skin Cancer.
• Colorectal Cancer.
• Cutaneous Metastases.
• Fasciocutaneous Flaps.

## 1 Introduction

Metastases represent one of the most outstanding characteristics of malignant neoplasms and are relatively rare in the skin, in spite of the great extension of the cutaneous organ [1]. They occur from 0.7% to 5% of patients with cancer. In those with metastatic disease, this rate is up to 10.4% [2]. Cutaneous metastases generally represent a late event in the course of an advanced internal malignancy with involvement of other organs, However, frequently it can be the first signal of malignancy, which occurs with greater frequency in lung carcinoma, followed by kidney and ovary cancer [1].

Of all tumors, breast cancer most commonly spreads as cutaneous metastasis by direct, hematogenic, and lymphatic pathways for spreading, with incidence of 24% according to a meta-analysis by Krathen [2].

Lung, colorectal, renal, ovarian and bladder cancer have similar rates of cutaneous metastases, which vary from 3.4% to 4%, mainly through blood and lymphatic dissemination [3].

Development of cutaneous metastases from colon cancer is a rare event, usually occurring in widely disseminated disease and commonly leading to a poor prognosis.

As to location, cutaneous metastases often favor areas close to the primary malignancy, such as lung cancer and skin metastases on the trunk. However, remote sites as the scalp may be also involved [1].

We present the case of a 92-year-old female patient with a single nodular skin lesion on her left supraclavicular area.

The work has been reported in line with the SCARE criteria [19].

## 2 Case report

A 92-year-old neglected female patient, living on her own, presented to our Department with a large ulcerated nodule on the left supraclavicular region that had been present for 10 months, growing progressively and consistently.

**Fig. 1:**
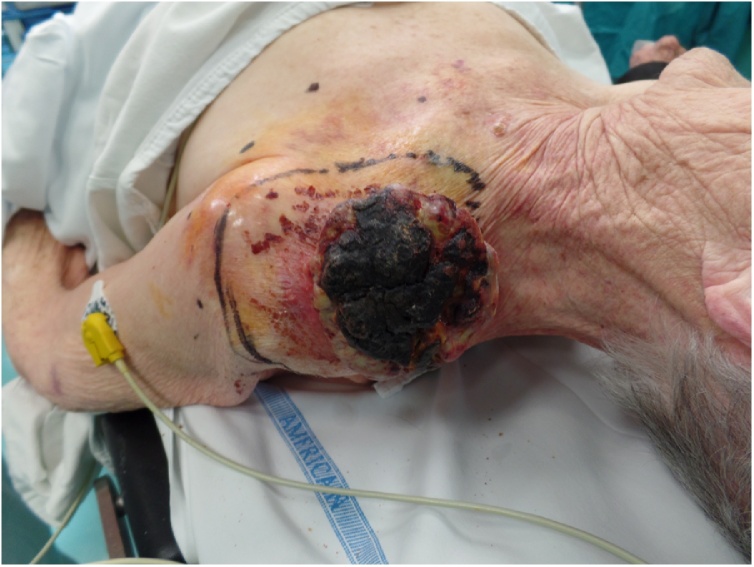
Clinical presentation of the tumor.

Physical examination showed a patient in poor general conditions with a 11 × 8 cm, ulcerated, cauliflower-like, with polilobulated margins, nodule in her left supraclavicular region. The lesion appeared to invade the clavicle bone and cervical lymphnodes were not palpable (Fig. 1).

A punch biopsy was performed in order to assess the type of cutaneous malignancy, but unfortunately came back not diriment because of the large amount of necrotic tissue with rare isolated keratin pearl.

**Fig. 2:**
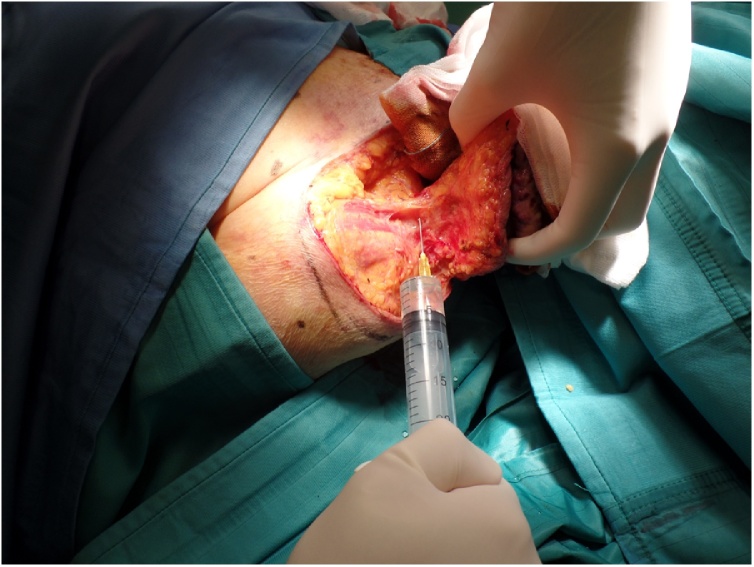
Intraoperative view of the deep aspect of the tumor invading the clavicle bone.

Then we decided to perform a surgical excision with two centimeters margins en-bloc with the periosteum of the clavicle, to which the lesion appeared to be adherent, under local anesthesia and sedation (Fig. 2).

The loss of tissue substance was then reconstructed by direct closure performing a wide undermining of the wound margins in a subfascial plane, under the fascia of the pectoralis major muscle anteriorly and of the trapezius muscle posteriorly.

**Fig. 3:**
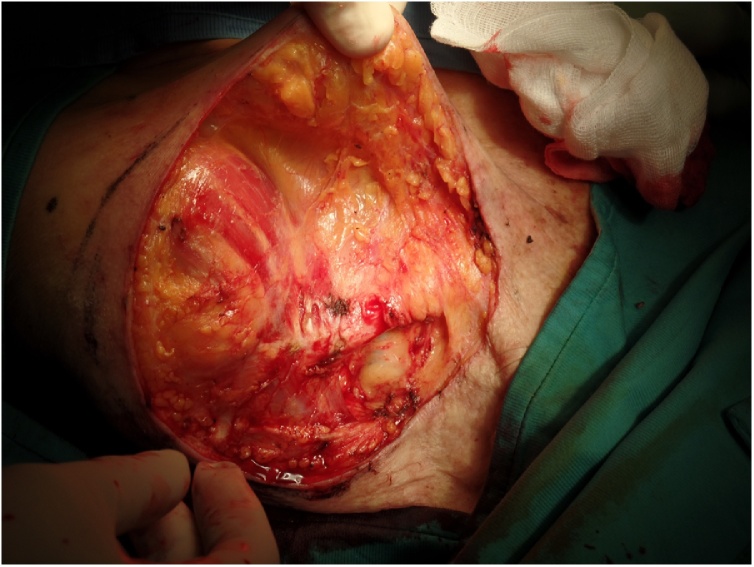
Intraoperative view of the harvesting of two fasciocutaneous flaps.

The choice of including a fascia in the direct closure of the wound has been made in order to give a better coverage to the clavicle bone, whose periosteum was previously removed (Fig. 3).

**Fig. 4:**
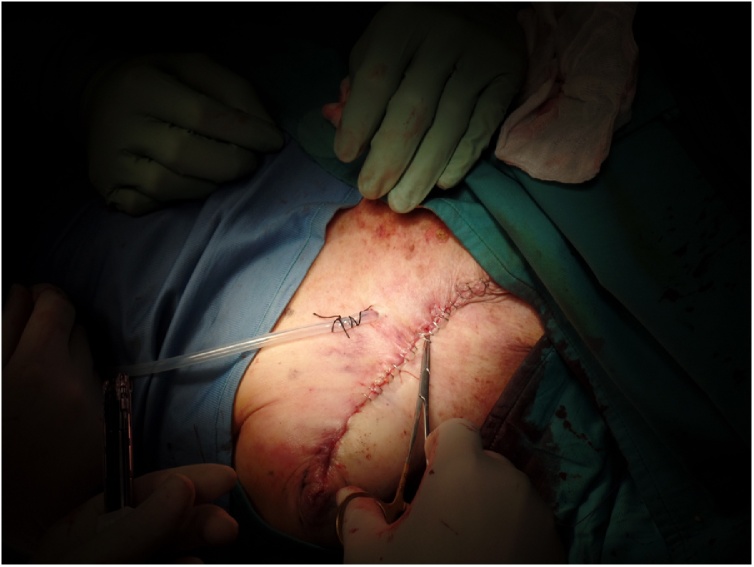
The wound closed by layers at the end of the surgical procedure.

A drain tube was positioned and the surgical wound was closed by direct suture (Fig. 4).

The postoperative period was uneventfully, the drain tube was removed after 4 days and stitches was removed in two weeks.

**Fig. 5:**
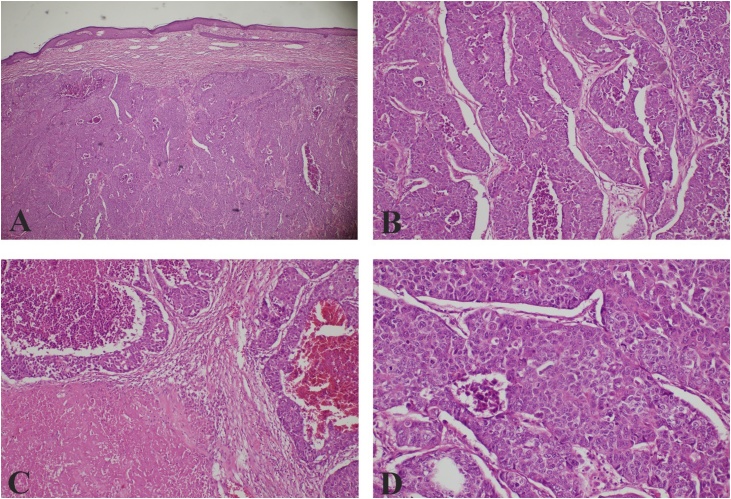
Dermal infiltration by neoplastic proliferation with pushing margins (A); this neoplastic proliferation has a solid, trabecular and pseudoglandular growth pattern (B), with focal areas of comedonecrosis and extensive areas of coagulative necrosis and hemorragia (C); the tumor cells are large with abundant eosinophilic cytoplasm and nuclei with finely dispersed chromatin and prominent nucleoli, there are many atypical mitotic figures (D). Hematoxylin-eosin stain.

**Fig. 6:**
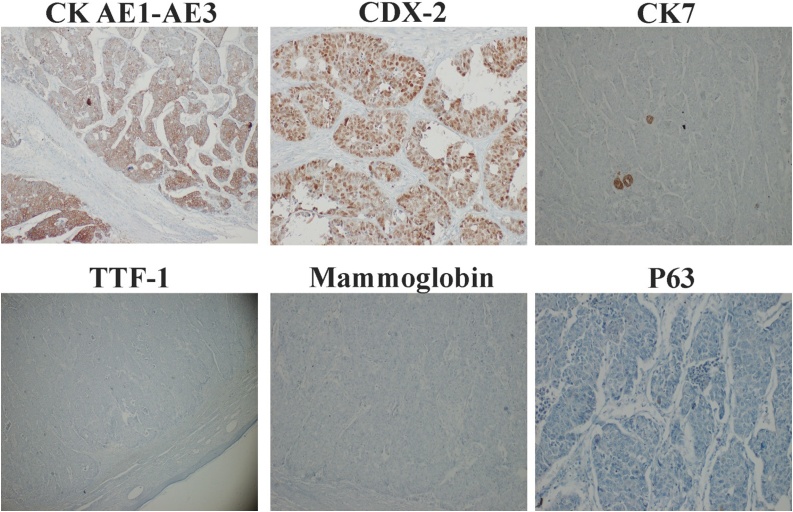
Immunohistochemistry stains; the neoplastic proliferation showed positive stain for CK AE1-AE3 and CDX2, highlighting an epithelial differentiation and likely origin from large intestine. The tumor cells showed negative stain for TTF-1, CK7, Mammoglobin, P63, neuroendocrin markers (CD56, sinaptofisina) and S100.

The histopathological examination revealed large tumor cells with abundant eosinophilic cytoplasm and nuclei with finely dispersed chromatin and prominent nucleoli. Immunohistochemistry revealed positive stain for CK AE1-AE3 and CDX2, highlighting an epithelial differentiation and likely origin from large intestine. The tumor cells showed negative stain for TTF-1, CK7, Mammoglobin, P63, neuroendocrin markers (CD56, sinaptofisina) and S100 (Fig. 5, Fig. 6). Final diagnosis was a cutaneous metastasis from an occult adenocarcinoma of the colon. All the resection margins were clear.

**Fig. 7:**
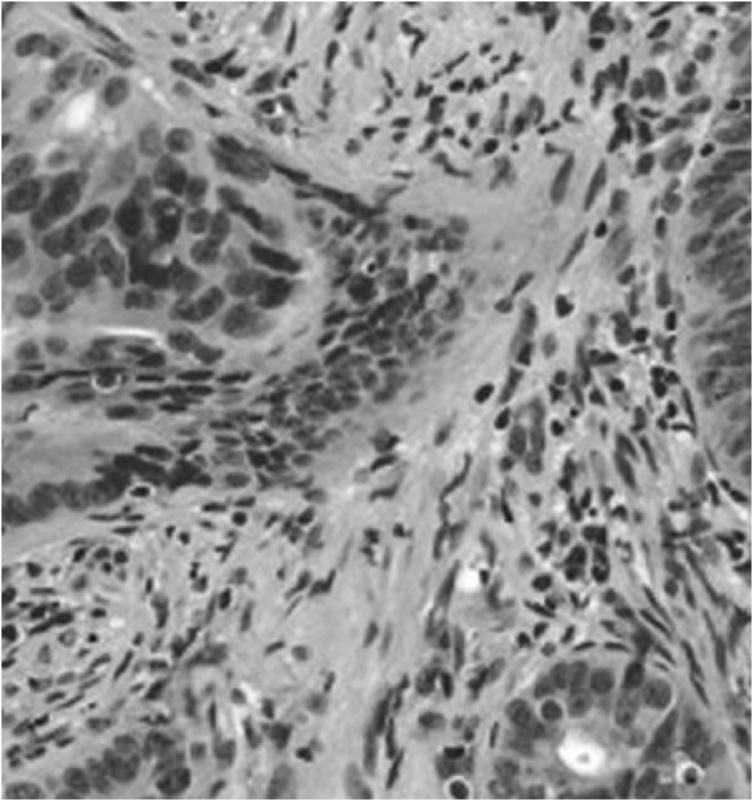
Punch biopsy came back positive for moderately differentiated adenocarcinoma composed by cells with abundant cytoplasm, nuclei with dispersed chromatin and prominent nucleoli. Hematoxylin-eosin stain.

**Fig. 8:**
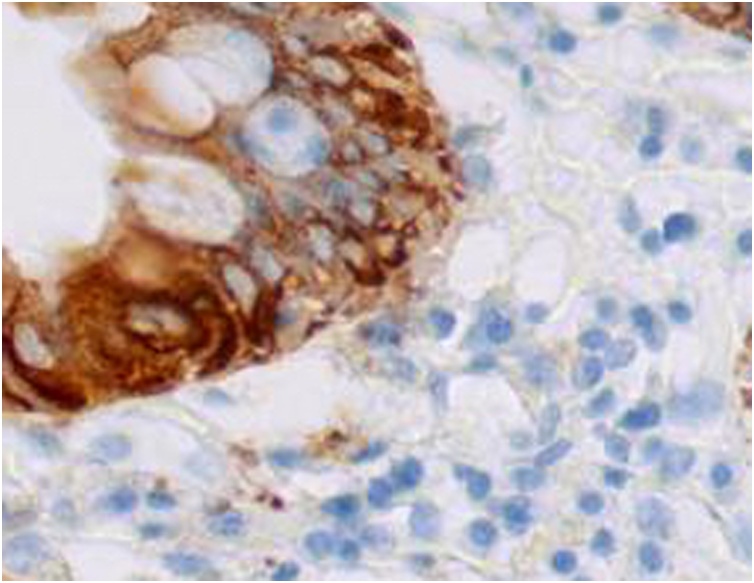
Immunohistochemistry stains of the punch biopsy; the neoplastic proliferation showed positive stain for CK AE1-AE3.

Two weeks after the surgery, a colonoscopy was scheduled and a large vegetative mass of the descending colon was found. A punch biopsy was performed that came back positive for moderately differentiated adenocarcinoma composed by cells with abundant cytoplasm, nuclei with dispersed chromatin and prominent nucleoli (Fig. 7) positive stain CK AE1-AE3 and CDX2 (Fig. 8).

We then programmed a PET scan one month after surgery to assess the presence of the primary tumor, but unfortunately the patient died due to a myocardial infarction.

## 3 Discussion

Cutaneous metastasis of colorectal adenocarcinoma a rare event (2.3%–6%) that usually occur two years after the detection or resection of the primary tumor [4,5]. It seldom occurs before the identification of the primary tumor and involvement of secondary organs, such as the liver. There are few cases reported with only cutaneous metastases [6,7].

The most frequent cutaneous site of colon cancer metastasis is the surgical scar in the abdomen that results from the removal of the malignancy. The metastasis may also occur in preexistent non-related surgical scars, but there are few cases reported [8,9]. Umbilical metastasis (“Sister Mary Joseph nodule”) may be a frequent finding. Other cutaneous sites, in descending order of frequency, are: pelvis, back, thorax, extremities, head and neck [10].

Skin cancer be expression either of a primary disease or of a secondary disease, in which the infiltration is due to a systemic disease or a disease recurrence [11].

Sometimes there is also a correlation between skin lesions and underlying cancers of different nature [12,13] or correlation with an higher risk in developing other tumors [14].

Several mechanisms of cutaneous metastasis have been postulated. Kauffman and Sina suggested that metastatic spread of adenocarcinoma to the skin and subcutaneous tissue could be caused by lymphatic and hematogenous spread, direct extension, or implantation during surgery [15].

In this report, the patient presented a single metastatic lesion located in the left shoulder area, probably through blood or lymphatic dissemination.

Clinical presentation of cutaneous metastases is quite variable. Lesions may be single or multiple, violaceous or skin-colored, hard or soft, may mimic epidermal cysts, neuro-fibromas or lipomas. More rarely, it may simulate infection, which is called inflammatory metastatic carcinoma or erysipeloid carcinoma. At the histological examination, the identification of the primary tumor is not always possible by the usual methods, because metastases are frequently more anaplastic [2,10].

The majority of metastases are well differentiated, mucin-secreting adenocarcinomas. They usually have a nodular morphology and are located in the dermis, with subsequent infiltration of epidermis and the subcutaneous tissue with a narrow area of the papillary dermis uninvolved by underlying pathology called Grenz zone.

Survival after cutaneous metastasis diagnosis varies from 1 to 34 months.

The average survival of patients after the diagnosis of cutaneous metastasis of colon carcinoma is 18 months [10].

Single cutaneous metastasis have to be surgically removed. For extensive cutaneous metastases, the treatment is only palliative, for they are linked to significantly higher rates of distant metastases and uncontrolled local disease, as well as lower survival rates [16].

In conclusion, dermatological evaluation of patients who are undergoing screening or who have already been diagnosed with cancer is extremely important, for it provides a high level of suspicion with the onset of cutaneous lesions, even if these are clinically compatible with benign illnesses – as the example of the present case, in which the cutaneous metastasis was single and mimicked a squamous cell carcinoma of the skin. This evaluation also helps with the therapy, because most of the times, cutaneous metastases already indicate a worse prognostic of a neoplastic disease.

For what concerns surgical treatment for cutaneous metastasis of non-skin malignancy, a wide excision with at least 1 centimenter margin is highly recommended in order to let the assessment of lymphovascular invasion by the pathologist.

In terms of reconstruction, direct closure, when feasible, is the preferred option because it gives the possibility to approximate the original margins of the excision.

If direct closure is not an option, skin grafts, both full- and split-thickness, are a valuable alternative but only on well vascularized wound bed, otherwise grafts do not take.

In this case, the periosteum investing the underlying clavicle bone was removed due to the tumor infiltration, so in order to achieve a well vascularized coverage we undermined the wound margin in a subfascial plane.

Is widely demonstrate how including the fascia increases blood supply in every type of skin flaps [17,18].

## Conflicts of interest

None.

## Funding

No funding.

## Ethical Approval

Ethical approval exempted by our institution.

## Consent

Written informed consent was obtained from the patient for publication of this case report and accompanying images. A copy of the written consent is available for review by the Editor-in-Chief of this journal on request.

## Author contribution

Mario Faenza: writing the paper

Giuseppe Del Torto: study concept

Pasquale Di Costanzo: data collection

Gorizio Pieretti: data collection

Rossella Lamberti :pathology report

Renato Franco: pathology report

Giuseppe A Ferraro: review of the literature

Giovanni F Nicoletti: study concept

## Registration of Research Studies

This case is not a first-in-man study.

## Guarantor

Mario Faenza, M.D.

## Provenance and peer review

Not commissioned, externally peer reviewed.

## References

[1] Rochael M.C., Estrella R.R., Neves R.G., Lupi O. (2001). Metástases cutâneas. Orgs. Câncer da Pele.

[2] Krathen R.A., Orengo I.F., Rosen T. (2003). Cutaneous metastasis: a meta-analysis of data. South. Med. J..

[3] Hu S.C., Chen G.S., Wu C.S., Chai C.Y., Chen W.T., Lan C.C. (2009). Rates of cutaneous metástases from different internal malignancies: experience from a Taiwanese medical center. J. Am. Acad. Dermatol..

[4] Reilly W.T., Nelson H., Schroeder G., Wieand H.S., Bolton J., O’Connell M.J. (1996). Wound recurrence following conventional treatment for colorectal cancer: a rare but perhaps underestimated problem. Dis. Colon Rectum.

[5] Saeed S., Keehn C.A., Morgan M.B. (2004). Cutaneous metastasis: a clinical, pathological, and immunohistochemical appraisal. J. Cutan. Pathol..

[6] Camci C., Türk H.M., Büyükberber S., Karakök M., Koruk M., Beyazity Y. (2002). Colon carcinoma with synchronous subcutaneous and osseous metastasis: a case report. J. Dermatol..

[7] Wright P.K., Jha M.K., Barrett P.D., Bain I.M. (2003). Colonic adenocarcinoma presenting as a cutaneous metastasis in an old operative scar. J. Postgrad. Med..

[8] Gupta S.S., Singh O. (2010). Carcinoma colon presenting as cutaneous metastasis to an old operative scar of hysterectomy. J. Cancer Res. Ther..

[9] Lookingbill D.P., Spangler N., Helm K.F. (1993). Cutaneous metastases in patients with metastatic carcinoma: a retrospective study of 4020 patients. J. Am. Acad. Dermatol..

[10] Nesseris I., Tsamakis C., Gregoriou S., Ditsos I., Christofidou E., Rigopoulos D. (2013). Cutaneous metastasis of colon adenocarcinoma: case report and review of the literature. An. Bras. Dermatol..

[11] Faenza M., Ronchi A., Santoriello A., Rubino C., Pieretti G., Guastafierro A., Ferraro G.A., Nicoletti G.F. (2017). What’s new on primary Hodgkin’s lymphoma of the breast? A case report and review of the literature. Int. J. Surg. Case Rep..

[12] Guastafierro A., Verdura V., Di Pace B., Faenza M., Rubino C. (2019). The influence of breast cancer on the distribution of cherry angiomas on the anterior thoracic wall: a case series study. Dermatology.

[13] Pileri A., Misciali C., Baraldi C., Sechi A., Faenza M., Fanti P.A., Stella A., Patrizi A. (2017). Erosive pustular dermatosis of the leg: an uncommon entity?. G. Ital. Dermatol. Venereol..

[14] Ricciardiello F., Caraglia M., Iorio B., Abate T., Boccellino M., Colella G., Oliva F., Ferrise P., Zappavigna S., Faenza M., Ferraro G.A., Sequino G., Nicoletti G.F., Mesolella M. (2017). Aggressiveness pattern and second primary tumor risk associated with basaloid squamous cell carcinoma of the larynx. Oncotarget.

[15] Kauffman C.L., Sina B. (1997). Metastatic in ammatory carcinoma of the rectum: tumor spread by three routes. Am. J. Dermatopathol..

[16] Gu Y., Tang R., Gong D.Q., Qian Y.L. (2008). Reconstruction of the abdominal wall by using a combination of the human acellular dermal matrix implant and an interpositional omentum flap after extensive tumor resection in patients with abdominal wall neoplasm: a preliminary result. World J. Gastroenterol..

[17] Rubino C., Faenza M., Di Pace B., Campitiello N., Brongo S., Zingone G. (2017). A new keystone flap "Plus" design: case series and analysis of follow-up. J. Plast. Reconstr. Aesthet. Surg..

[18] Faenza M., Pieretti G., Lamberti R., Di Costanzo P., Napoletano A., Di Martino M., Casale F., Ferraro G.A., Nicoletti G.F. (2017). Limberg fasciocutaneous transposition flap for the coverage of an exposed hip implant in patient affected by ewing sarcoma. Int. J. Surg. Case Rep..

[19] Agha R.A., Fowler A.J., Saetta A., Barai I., Rajmohan S., Orgill D.P., for the SCARE Group (2016). The SCARE statement: consensus-based surgical case report guidelines. Int. J. Surg..

